# Position Statement on Exercise Dosage in Rheumatic and Musculoskeletal Diseases: The Role of the IMPACT-RMD Toolkit

**DOI:** 10.31138/mjr.32.4.378

**Published:** 2021-12-27

**Authors:** George S. Metsios, Nina Brodin, Thea P.M. Vliet Vlieland, Cornelia H.M. Van den Ende, Antonios Stavropoulos-Kalinoglou, Ioannis Fatouros, Martin van der Esch, Sally A.M. Fenton, Katerina Tzika, Rikke Helene Moe, Jet J.C.S. Veldhuijzen van Zanten, Yiannis Koutedakis, Thijs Willem Swinnen, Aristidis S. Veskoukis, Carina Boström, Norelee Kennedy, Elena Nikiphorou, George E. Fragoulis, Karin Niedermann, George D. Kitas, The IMPACT-RMD Consortium

**Affiliations:** 1 School of Physical Education, Sport Science and Dietetics, Department of Nutrition and Dietetics, University of Thessaly, Greece,; 2 Department of Rheumatology, Russells Hall Hospital, Dudley Group NHS Foundation Trust, Dudley, United Kingdom,; 3 Faculty of Education, Health and Wellbeing, University of Wolverhampton, United Kingdom,; 4 Department of Neurobiology, Care Sciences and Society, Division of Physiotherapy, Karolinska Institutet, Huddinge, Sweden,; 5 Department of Orthopaedics, Rehabilitation and Physical Therapy, Leiden University Medical Center, Leiden, the Netherlands,; 6 Department of Rheumatology, Sint Maartenskliniek, Nijmegen, the Netherlands,; 7 Centre for Active Lifestyles, Carnegie School of Sport, Leeds Beckett University, Leeds, United Kingdom,; 8 Department of Physical Education and Sport Science, University of Thessaly, Karies, Trikala, Greece,; 9 ACHIEVE - Center of Applied Research, Faculty of Health, Amsterdam University of Applied Sciences. Reade, Center for Rehabilitation and Rheumatology/Amsterdam Rehabilitation Research Center, Amsterdam, The Netherlands,; 10 School of Sport, Exercise and Rehabilitation Sciences, University of Birmingham, Birmingham, United Kingdom,; 11 National Resource Centre for Rehabilitation in Rheumatology, Department of Rheumatology, Diakonhjemmet Hospital, Oslo, Norway,; 12 Division of Rheumatology, University Hospitals Leuven, Leuven, Belgium,; 13 School of Allied Health, Faculty of Education and Health Sciences and Health Research Institute, University of Limerick, Limerick, Ireland,; 14 Rheumatology Department, King’s College Hospital, London, United Kingdom,; 15 Institute of Infection, Immunity and Inflammation, University of Glasgow, Glasgow, United Kingdom,; 16 School of Health Professions, Institute of Physiotherapy, Zurich University of Applied Sciences, Winterthur, Switzerland

**Keywords:** Exercise, physical activity, rheumatic and muscoluskeletal disease, inflammatory arthritis, rheumatoid arthritis, osteoarthritis, inflammation

## Abstract

There is convincing evidence to suggest that exercise interventions can significantly improve disease-related outcomes as well as comorbidities in rheumatic and musculoskeletal diseases (RMDs). All exercise interventions should be appropriately defined by their dose, which comprises of two components: a) the FITT (frequency, intensity, time and type) and b) the training (ie, specificity, overload, progression, initial values, reversibility, and diminishing returns) principles. In the published RMD literature, exercise dosage is often misreported, which in “pharmaceutical treatment terms”, this would be the equivalent of receiving the wrong medication dosage. Lack of appropriately reporting exercise dosage in RMDs, therefore, results in limited clarity on the effects of exercise interventions on different outcomes while it also hinders reproducibility, generalisability and accuracy of research findings. Based on the collective but limited current knowledge, the main purpose of the present Position Statement is to provide specific guidance for RMD researchers to help improve the reporting of exercise dosage and help advance research into this important field of investigation. We also propose the use of the IMPACT-RMD toolkit, a tool that can be used in the design and reporting phase of every trial.

## INTRODUCTION

Rheumatic and musculoskeletal diseases (RMDs) are a group of non-communicable diseases characterised by debilitating symptoms, such as pain, fatigue, inflammation, and/or joint damage. These may result in subsequent loss in the range of motion and functional impairment in one or more areas of the musculoskeletal system. The most prevalent RMDs, based on the European Alliance of Associations for Rheumatology (EULAR) Position Statement, are osteoarthritis (OA), rheumatoid arthritis (RA), osteoporosis (not a classical RMD but still reported in the EULAR Position Statement), low back pain (an RMD symptom which is reported in the EULAR Position Statement), ankylosing spondylitis, psoriatic arthritis, gout, fibromyalgia, and connective tissue diseases, such as Systemic Lupus Erythematosus and systemic sclerosis. The management of RMDs involves a combination of multiple different pharmaceutical and non-pharmaceutical interventions. The introduction of modern but still expensive treatments, such as biological disease modifying antirheumatic drugs (bDMARDs), has had a significant effect at improving outcomes in some RMDs but has also increased substantially healthcare costs. Despite the improvements in pharmaceutical therapies, many RMD patients do not reach complete remission and may still experience eventually variable levels of disability. For example, 20% to 40% of patients treated with a tumour necrosis factor alpha inhibitor fail to achieve even an ACR20 response (ie, a 20% improvement as calculated using the American College of Rheumatology [ACR] criteria^1^), while some patients lose response over time and/or experience adverse events.^1^ Also, for many RMDs there is no effective drug treatment available, eg, from the very common OA to the rare systemic sclerosis. These facts suggest that there is a measurable therapeutic void in RMDs while at the same time, RMD treatment costs remain high. It is necessary, therefore, that the overall management of RMDs should also focus on adjunct non-pharmacological interventions with proven effectiveness, in order to improve quality of life and reduce, prevent or help to better cope (physically, emotionally and societally) with the activity limitations and adverse effects of suffering from an RMD.

A promising and well-researched intervention that can significantly improve symptoms and quality of life in non-communicable diseases^2^ including RMDs,3 is physical activity and/or exercise. Physical activity is any bodily movement that increases energy expenditure above resting levels (such as gardening or doing household chores). Exercise on the other hand, is a distinct part of physical activity, which is a structured and planned behaviour (such as going swimming two times per week) that aims to improve specific outcomes, such as cardiorespiratory fitness, fatigue, or activity limitation. Our knowledge on the effects of exercise on RMD symptoms has significantly improved in the last 30 years. Despite earlier clinical practice approaches to avoid exercise in fear of exacerbating disease symptoms, exercise is now considered an important intervention for improving symptoms in RMDs, to the point that the EULAR is actively promoting the use of exercise and physical activity in recent recommendations^3,4^ and promotes the implementation of physical activity at scale.^5^ This is because collective results suggest that exercise can have beneficial effects in multiple different patient-relevant and clinically-important outcomes in RMDs. For example, in osteoarthritis exercise can reduce pain^6^ and cardiovascular disease risk^7,8^ and improve quality of life^9^; in RA, exercise improves cardiorespiratory fitness and functional ability,^10–12^ reduces pain and fatigue,^11,13,14^ can increase muscle mass and reduce adiposity^15–17^ more than bDMARDs,^18^ thus reversing the highly prevalent rheumatoid cachexia^19,20^; in fibromyalgia exercise is the only intervention that can be recommended by EULAR for therapy (based on the most recent meta-analysis) with strong evidence for its effectiveness.^21^ Moreover, exercise interventions improve health-related outcomes in a dose-dependent manner; this suggests that more exercise or higher intensities during exercise may result in better improvements, while depending on how the exercise dosage is applied, the magnitude and the time-course of these beneficial adaptations can vary.^2^

From a theoretical perspective, exercise should be prescribed in the same way as medication, where specific symptoms are taken into account in combination with specific clinically-important cut-off points. However, methodological limitations around the dosage of exercise that we frequently see in RMD exercise trials, hinder our precise understanding about the effects of different exercise dosages as well as their dose-dependent effects on different RMD outcomes.

The IMPACT-RMD is a group of international experts and patient organizations from seven EU countries with an extensive research track record in RMDs and contributions in EULAR recommendations,^4,22^ including the recent EULAR physical activity recommendations.^3^ The IMPACT-RMD Consortium, is also a funded EULAR initiave, responsible for implementing physical activity and exercise in clinical practice.^5^ The Consortium has identified that research in RMDs is characterized by inconsistencies in reporting exercise dosage, and therefore has developed this Position Statement (based on roundtable discussions) to highlight issues around exercise dosage in RMDs, present the evidence on the effects of exercise dosage on different outcomes in RMDs as well as to provide a guide/toolkit for appropriate designing and reporting exercise dosage in RMD trials.

## DEFINING THE DOSAGE IN PHYSICAL ACTIVITY AND EXERCISE INTERVENTIONS

Given the lack of reporting exercise dosage accurately in RMD research trials, future exercise RMD research should aim to improve the reporting of two distinct set of principles: a) the exercise principles and b) the training principles. Both sets of principles need to be accurately reported in exercise trials so that we can: a) accurately interpret the overall effects (and dose-response) of exercise protocols on the potential benefits as well as harms, b) the opportunities and challenges for their large scale application in different settings and c) their specificity in terms of the different RMD populations.

### Exercise Principles

The definition of dosage in any exercise intervention, irrespective of the population studied, is the process of providing information on four specific exercising principles: Frequency, Intensity, Time and Type (F.I.T.T.).^23^ In exercise physiology research, these are collectively referred to as “the FITT principles” and these need to be mentioned within the methodology of every exercise intervention (**Table 1**). If this is not the case, the dosage of exercise and its cause-and-effect and dose-dependent relationship with health-related outcomes cannot be determined while research methodologies cannot be reproduced. There have been recent attempts to improve the reporting of the goals and the content of exercise interventions with regards to the FITT principles,^24,25^ however, these have not been taken-up effectively and systematically in exercise RMD research.

**Table 1.** The IMPACT-RMD toolkit: appropriate reporting of exercise dosage using the FITT and training principles.

**Exercise Dosage**	**Item**	**Checklist item with Example**	**Tick if reported**
**FITT PRINCIPLES**			
Frequency	1	Report the exact baseline (ie, at the start) frequency of the exercise/physical activity program. This is how many times per week the program takes place. EXAMPLES: 1. The baseline exercise frequency of the aerobic exercise training program was three times/week. 2. The baseline exercise frequency of the strength/resistance exercise training program was once a week.	
Intensity	2	Report the exact baseline exercise intensity of the exercise/physical activity program. The intensity can be reported in many different ways, eg, percentages (%) of heart rate maximum and heart rate reserve, or % of maximal oxygen uptake, or rate of perceived exertion (RPE) for patients with RMDs that may have a cardiovascular condition (eg, arrhythmias, defibrillator or b-blockers). EXAMPLES: 1. The baseline exercise intensity of the aerobic exercise training program was a heart rate corresponding to 70% of maximal oxygen uptake, achieved during the cardiorespiratory fitness testing. 2. The baseline exercise intensity of the strength / resistance exercise training program was 60% of the one maximum repetition.	
Time	3	Report the exact baseline time of the exercise / physical activity program. This is how long each exercise session lasts. Details on the components of each session (warm-up, conditioning, cool-down) is needed. Also, the total duration of the programme (usually in weeks) should be reported EXAMPLES: 1. The baseline exercise time of each aerobic exercise session was 45 minutes, from which 10 minutes were dedicated for warm-up and 10 minutes for cool-down. 2. The baseline exercise time of each strength / resistance exercise session was 30 minutes, from which 10 minutes were dedicated for warm-up and 5 minutes for cool-down. **Note**: specific information for the warm-up and cool-down exercises should also be reported.	
Type	4	Report the exact type of the exercise intervention. EXAMPLES: **Warm-up**: 2min on the bike (40% HRmax), stretching (*exercises need to be defined*). **Cool-down:** 5 minutes of slow walking and stretching (*exercises need to be defined*). **Example aerobic exercise session**: Interval training using aerobic exercises, and specifically walking (treadmill), cycling (cycle ergometer) and rowing (rowing ergometer). All these three aerobic exercises formed one circuit. In each circuit, patients exercised for 3 minutes in each of these modes of exercise, followed by 1 minute of active resting (ie, slow walking at 40% of HRmax). All patients performed three circuits in each aerobic training exercise session. **Example resistance training exercise session:** The program consisted of exercises in 5 large muscle groups (*the muscle groups and exercises should be explicitly reported*). For each muscle group, participants performed 3 sets of exercises each with 8–12 repetitions. Each repetition lasted 1/1 sec (ie, 1 sec for the concentric and 1 sec for the eccentric phase). The rest between the sets was 30 sec, while between muscle group exercises was 60 sec.	
**EXERCISE TRAINING PRINCIPLES**			
Specificity	5	The training adaptations must be specific to the organ system or muscles trained with exercise. EXAMPLES: 1. Interval aerobic training was utilized for improving the primary outcome, ie, cardiorespiratory fitness. 2. Strength / resistance training was used to increase muscle mass which was the primary outcome of the present study.	
Progression	6	Over time, the body adapts to exercise. For continued improvement, elements of the FITT principles must be adjusted accordingly (eg, intensity). EXAMPLES: 1. The intensity of the high intensity interval training (HIIT) program was increased after the first month of training from 85% of maximum heart rate to 90% of maximum heart rate for the following month. 2. The frequency of the strength / resistance training program was increased after the first month of training from once a week to twice a week for the remaining 2 months.	
Overload	7	For an intervention to improve fitness or strength, the training volume must exceed current habitual physical activity and/or strength training levels. These levels can be assessed at baseline and/or incorporated in the eligibility criteria (inclusion and exclusion of participants) EXAMPLES: 1. Baseline physical activity levels from the present sample of patients suggested that they were physical inactive (*provide physical activity levels or cardiorespiratory fitness levels at baseline*). 2. Exclusion criteria: we excluded patients that were currently taking part or took part for the last year, within any strength training program.	
Initial values	8	Improvements in the outcomes of interest will be greatest in those with lower initial values. Thus, recording and acknowledging baseline values is highly recommended. EXAMPLES as above in Item 7.	
Reversibility	9	Once a training stimulus is removed, fitness levels will eventually diminish and return to baseline. This is particularly important for studies with cross-over designs (where the exercise stimulus is removed after the exercise group is crossing over to the other arm of the study) or follow-up studies (ie, follow-up after the end of the intervention). This is almost a synonym with “detraining, ie, what happens to the human body once the exercise training stimulus stops. EXAMPLE: Patients were followed up for 1 month after the exercise intervention stopped, to investigate the detraining effects (ie, how the primary outcome changed after the exercise stimulus was removed)	
Diminishing returns	10	The expected degree of improvement in fitness decreases as individuals become more fit, thereby increasing the effort required for further improvements. As such, any adjustments in the FITT principles should be clearly planned and stated in any research study. EXAMPLES as in Item 6 (Progression).	

### Training Principles

**Figure 1.**
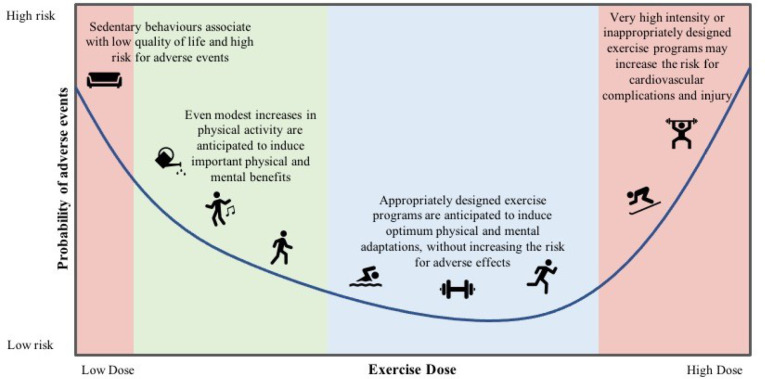
Theoretical U-Shaped relationship between exercise dosage and health-related outcomes in RMDs.

The FITT principles are not the only important piece of information that should be explicitly described in exercise trials. To improve the reporting of exercise dosage, researchers should also have the responsibility to consider, embed, report accurately and consistently the established exercise training principles which are *specificity, overload, progression, initial values, reversibility* and *diminishing returns* (details for all the exercise training principles with examples, appear in **Table 1**).^23^ Implementing the training principles in exercise interventions ensures that the specifics of the exercise intervention are appropriately planned and delivered, so that the benefits of such interventions can be optimized for the receiving patient. It is worth mentioning that researchers predominantly support the use of the FITT exercise principles for determining dosage^24,25^ while efforts to develop interventions that appropriately embed the training principles are lacking. However, considering and appropriately implementing the training principles in exercise interventions is of crucial importance in RMDs. Engaging in high intensity exercise, particularly at the start of an exercise program – required to improve cardiovascular health and reduce fatigue,^13^ pain,^11,26^ or activity limitation^10^ – may be problematic in people with RMDs, as early application of higher intensities may result in injury or cardiovascular complications.^27^ For example, if we consider the training principle “progression”, a detailed description should be included in a research trial on the initial intensity of the program (ie, what % of intensity was used during the first set of exercise sessions) and how this progressively increased to achieve the targeted intensity of the exercise program (eg, the 85% of the heart rate reserve). It is difficult to believe that patients with RMD could start exercising at 85% of their heart rate reserve during their first set of exercise sessions, yet most exercise trials do not provide information on whether initial sessions (ie, during the first weeks of applying the exercise program) with familiarisation took place at the initial stages of the intervention. Issues with lack of appropriately reporting the FITT principles and the remaining training principles further contribute to the inconsistencies that characterise this research area. **Figure 1** represents a theoretical approach of applying the exercise dosage when exercise is provided for RMDs.

## EFFECTS OF STUDIED EXERCISE DOSAGE IN RHEUMATIC AND MUSCULOSKELETAL DISEASES

**Figure 2.**
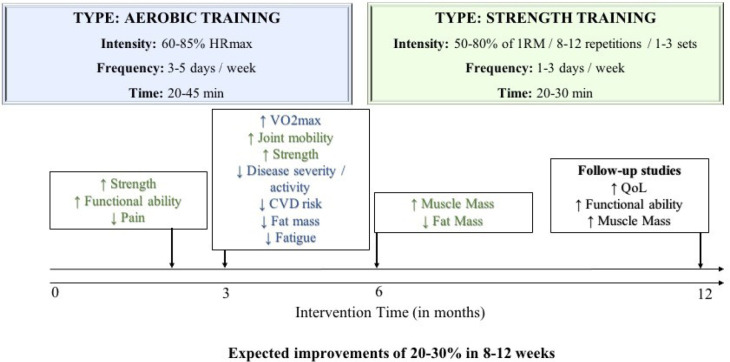
Expected aerobic (blue) and strength (green) training adaptations in RMDs.

The lack of appropriately reporting the FITT and training principles in RMD research, is also observed in other non-communicable diseases such as cancer^28^ and stroke.^29^ Not surprisingly, these issues also characterise the studies that have formed the EULAR 2018 recommendations.^3^ The current recommendations about exercise dosage can be collectively summarised, as follows:

- *Aerobic exercise interventions*: Aerobic training exercise interventions that utilise intensities of 60–85% of maximum heart rate, a frequency of 3–5 days/week while the time of the exercise session is 20–45 min, elicit significant improvements in multiple health-related outcomes, as early as three months (**Figure 2**).
- *Strength training interventions*: When the intensity of a strength training program is 50–80% of 1 repetition maximum, each exercise is performed for 8–12 repetitions per exercise and 1–3 sets per exercise, the frequency is 1–3 days / week and the time for the strength training session is 20–30 min, then significant improvements in different outcomes are observed in as early as two months (**Figure 2**).

Interventions that combine types of exercise (for example, combined aerobic and strength training) demonstrate similar results, ie, expected improvements appear at the same time points as with aerobic and/or resistance exercise alone,^12,30^ albeit, these can vary according to the dosage applied. Moreover, studies that have followed-up people with RMD post intervention, reveal that improvements in quality of life, strength and functional ability are maintained up to six months post exercise intervention.^31^

However, it is important to note, that even if details of these exercise interventions exist, the FITT principles are still too wide (ie, aerobic: 60–85% of heart rate reserve and strength: 50–80% of 1 repetition maximum) while the training principles are not reported. For example, 60% of heart rate reserve is considered moderate intensity while 85% high intensity; those two different intensities will result in physiological changes of different magnitude given the dose-dependent relationship of exercise with health outcomes. In “pharmaceutical treatment terms” this would be the equivalent of receiving the wrong medication dosage.

Within these limitations of reporting exercise and exercise training principles in RMD trials, the different intensities that have been utilised so far in RMD healthcare exercise research do not report exercise-related adverse outcomes and/or harms. Despite that the reporting of potential adverse effects of harms should be appropriately planned within the methodology of an exercise research trial, the current collective evidence suggests that exercise is considered safe in RMDs^11,32^ even with high intensity at a level of 80–90% of maximal oxygen uptake.^33^

## A TOOLKIT FOR METHODOLOGY PLANNING AND APPROPRIATE REPORTING OF EXERCISE DOSAGE IN RESEARCH TRIALS

The purpose of this Position Statement was to put together and highlight the elements of exercise programs that should be reported in RMD exercise trials. The FITT as well as the training principles exist for more than 30 years and have been the core of exercise training in athletes. However, RMD exercise trials lack this information, possibly due to different reasons, including lack of knowledge of their existence and/or the lack of a guide on what should actually be reported in exercise trials. To improve this area of research and enhance the understanding of the research and healthcare communities about the dosage of exercise and its effects on different outcomes, we urge future researchers to use the IMPACT-RMD toolkit (**Table 1**), in the design phase and reporting of future trials. The IMPACT-RMD toolkit summarizes all the elements of an exercise program, that could be used from the conception of the research hypothesis and designing of the exercise program (and later in the reporting) to the reporting of the exercise program details in the publication phase. It also provides examples for each of these elements, in order to aid the researchers to understand the exact details that are required within each FITT and training principle.

## DISCUSSION

The purpose of this Position Statement is to provide specific guidance for RMD researchers to help improve the reporting of exercise dosage and help advance research into this important field. To do this, we propose the use of the IMPACT-RMD toolkit, a tool which can facilitate significant improvement in this area of research. Significant issues still remain in RMD exercise research with regards to reporting dosage, while evidence from existing trials reveals that exercise – using a wide range of dosages – can significantly improve disease-related symptoms and comorbidities in RMDs.

In our approach to better understand the effects of specific dosages on specific RMD outcomes, it is important to note that RMD exercise research has almost exclusively focused on recruiting patients whose disease is well-controlled on their current medication^3^; even in these patients, there is uncertainty about the effects of different exercise dosages on different patient-and clinically-important outcomes. With regards to exercise engagement, more uncertainty exists for RMDs patients that are difficult to treat (eg, those with persistent pain and fatigue), because there is no evidence as to whether these patients should engage in exercise and if they do, what would be the appropriate dosage that will result in benefits without negatively affecting disease outcomes. Despite the positive steps that have been made in exercise RMD research trials in the last 30 years, the exact dosage of exercise interventions and exactly how, when and with what precautions it is applied to different RMD populations (and within each RMD at the different stages), is an area of research that requires further attention and improvement. The current lack of clarity in this research area, results in a prolongation of misreporting exercise dosage, the misunderstanding about adaptation specificity (benefits and harms) as well as the continuation of publishing data which lack precision thereby, affecting reproducibility. Investigating and thus, understanding the dose-dependent effects of exercise dosage on different outcomes, can potentially enhance the overall management of RMDs with benefical effects on the patients’ overall health and quality of life. Moreover, accurately reporting exercise dosage can enhance the accuracy, reproducibility and generalisability of any findings, and more importantly the opportunity for larger scale application of exercise protocols outside the clinical arena, and this can only be improved by enhancing our ability to interpret exactly what was delivered to what patient. Exercise (FITT) principles and training principles should be appropriately considered and implemented in the design phase, and accurately reported in detail in every future physical activity and exercise trial in RMDs. The IMPACT-RMD toolkit provided herein may significantly facilitate this effort.

## CONCLUSION

Exercise can have significant beneficial effects on RMD outcomes. Understanding the effects of specific exercises doses on health outcomes in RMDs is an area of research that requires improvement, so that frontline healthcare practitioners can confidently utilise and tailor different exercise interventions for improving RMD outcomes. The IMPACT-RMD toolkit can facilitate this process and help exercise RMD researchers plan, develop, implement and report accurately exercise dosage in relevant trials while it is also a tool that can support best practice in exercise prescription.
